# The Role of Innate and Adaptive Immune Cells in the Pathogenesis and Development of the Inflammatory Response in Ulcerative Colitis

**DOI:** 10.3390/jcm11020400

**Published:** 2022-01-13

**Authors:** Aleksandra Kałużna, Paweł Olczyk, Katarzyna Komosińska-Vassev

**Affiliations:** 1 Department of Clinical Chemistry and Laboratory Diagnostics, Faculty of Pharmaceutical Sciences in Sosnowiec, Medical University of Silesia in Katowice, 41-200 Sosnowiec, Poland; kvassev@sum.edu.pl; 2 Department of Community Pharmacy, Faculty of Pharmaceutical Sciences in Sosnowiec, Medical University of Silesia in Katowice, 41-200 Sosnowiec, Poland; polczyk@sum.edu.pl

**Keywords:** ulcerative colitis, lymphocytes, macrophages, dendritic cells, innate lymphoid cells

## Abstract

Ulcerative colitis (UC) is a chronic inflammatory disease with an underlying excessive immune response directed against resident microbiota and/or dietary antigens. Both innate and adaptive immune cells play a crucial role in the pathogenesis of UC. In the case of innate immune response cells, neutrophils, dendritic cells, macrophages have a crucial impact on the development of the disease, as well as innate lymphoid cells, which have received a particular attention in recent years. On the other hand, mechanisms of the adaptive immune response involve cells such as: cytotoxic lymphocytes, regulatory lymphocytes Treg, or helper lymphocytes Th–Th2, Th9, Th17, Th22, among which significant discoveries about Th9 and Th17 lymphocytes have been made in recent years. Due to the presence of antibodies directed against resident microbiota or one’s own tissues, the influence of B lymphocytes on the development of UC is also highlighted. Additionally, the impact of cytokines on shaping the immune response as well as sustaining inflammation seems to be crucial. This review briefly describes the current state of knowledge about the involvement of the innate and adaptive immune systems in the pathogenesis of UC. The review is based on personal selection of literature that were retrieved by a selective search in PubMed using the terms “ulcerative colitis” and “pathogenesis of ulcerative colitis”. It included systematic reviews, meta-analyses and clinical trials. Our knowledge of the involvement of the immune system in the pathophysiology of IBD has advanced rapidly over the last two decades, leading to the development of several immune-targeted treatments with a biological source, known as biologic agents.

## 1. Introduction

**Figure 1:**
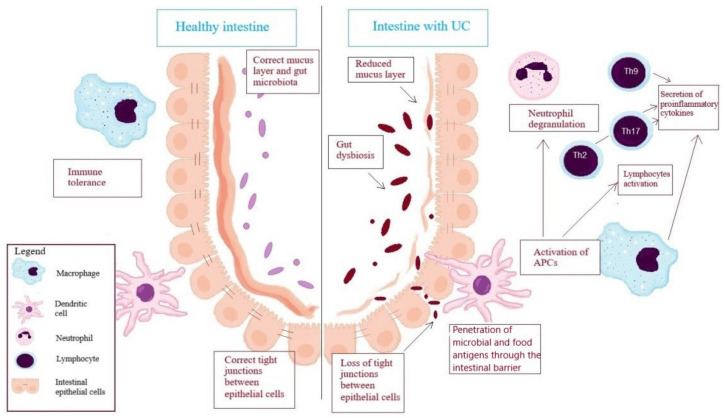
Pathogenesis of ulcerative colitis (UC). APCs—antigen presenting cells; In physiological conditions, a homeostasis between natural microbiota and immune system is maintained. Intestinal mucosa is covered by a thick layer of mucus preventing the penetration of intestinal bacteria to epithelial cells. Integrity of the intestinal barrier is provided by the tight junctions between epithelial cells, and the immune cells present a state of immune tolerance. In UC, a layer of mucus is reduced and the gut microbiota is abnormal, which leads to gut dysbiosis. A reduction of butyrate-producing bacteria (as a result of, e.g., antibiotic treatment) contributes to a decreased amount of butyrate in the intestinal lumen and enhances anaerobic metabolic processes, which generate less energy than the aerobic ones. The resulting oxidative stress damages the mucus-secreting colonocytes. Additionally, enhanced anaerobic processes favor the expansion of facultative anaerobes such as *Salmonella enterica* and *Escherichia coli*. Increased relative abundance of these bacterial species releasing bacterial enterotoxins directly attack and damage the intestinal epithelial cells, which in turn damages the intestinal mucosa and reduces its protective functions. The loss of tight junctions seen in UC allows bacteria and dietary antigens to pass through the intestinal barrier and leads to the activation of antigen-presenting cells. Macrophages and dendritic cells enhance neutrophil migration and degranulation, as well as activating lymphocytes Th2, Th17, Th9. Activation of immune cells results in the secretion of pro-inflammatory cytokines, i.e., TNF-α, IFN-γ, and IL-13, which increases the permeability of the intestinal barrier and thus promotes inflammation in the intestinal mucosa [3,4,5].

Ulcerative colitis (UC) is a chronic inflammatory disease, which belongs with Crohn’s disease to the group of the inflammatory bowel diseases (IBDs). The condition is diagnosed mostly between the ages of 20 to 40, however it can occur at every age. Characteristic of UC are alternating periods of clinical relapse and remission. Inflammation of the colon mucosa plays an essential role in pathogenesis of UC, which leads to ulcer formation. Changes observed within the intestinal mucosa are localized in the rectum and spread proximally to the other parts of the colon. The most common clinical symptoms are gastrointestinal disorders such as abdominal pain, diarrhea with mucus and/or blood, nausea and vomiting; nevertheless, general symptoms including fever, weight loss and anemia are also observed with the parenteral symptoms—peripheral arthritis, cholangitis, pyoderma gangrenosum, erythema nodosum and arthropathies [1,2]. Although the disorder is quite common, its exact pathogenesis is not fully understood; however, it is known that UC is connected with the excessive immune response to the environmental factors or resident microbiota among genetically susceptible subjects, and the immunity status plays a crucial role in the increased intestinal permeability and impaired barrier function, as presented graphically in Figure 1. There are reasonable doubts if the impaired barrier function precedes the onset of the disease, or the inflammation development in the lamina propria of the intestinal mucosa induces the intestinal epithelial disfunction [3].

Patients with UC present a decreased number of the goblet cells responsible, among others, for the excretion of biologically active substances that contribute to innate immunity, such as trefoil peptides and peptides binding Fc region of antibodies, as well as for secretion of the mucin, which forms large net-like polymers that lubricate the lumen and form a barrier between sterile intestinal epithelial and gut microbiota [6]. An impaired intestinal barrier and a decreased amount of the mucus leads to increased exposure to food antigens and antigens associated with gut microbiota, which activates the mechanisms of the innate immunity. Innate (passive) immune response is the first line of defense in the body, including cells such as macrophages, innate lymphoid cells, mast cells, and neutrophils to identify and eliminate pathogens in a short time, though its mechanisms are not specific for various antigens [5]. Antigen-presenting cells (APC)—macrophages and dendritic cells (DC)—connect the mechanisms of innate and adaptive (active) immune responses through presentation of the antigens to certain lymphocytes, which leads to activation of the adaptive immune cells. Presence of a specific cytokine environment results in the activation of transcription factors responsible for polarization of the naive lymphocytes, i.e., Th2, Th17, and Th9, playing crucial roles in the development of the disorder. Moreover, Treg cells, in spite of their increased number in the intestinal mucosa of patients with UC, present reduced immunoregulatory activity. Increased concentration of tumor growth factor β (TGF-β) induces the suppression of Th22 lymphocytes which secrete IL-10 with a protective effect on the intestinal mucosa. The impaired balance between pro- and anti-inflammatory processes leads to the development of the chronic inflammation and impairment of the intestinal barrier function. A key role in the development of the disease is played by cytokines whose functions and influence on inflammation are presented in Table 1 [5].

The altered balance of anti-inflammatory and pro-inflammatory cytokines (mainly towards the secretion of pro-inflammatory cytokines) may lead to the development of an excessive immune response, which is associated with further activation and infiltration of immune cells, increased cell apoptosis, and loss of intestinal barrier integrity [15].

## 2. Immune Cells in the Pathogenesis of Ulcerative Colitis

### 2.1. Cells of Innate Immunity

Immune cells play a significant role in the pathogenesis of ulcerative colitis, including cells of innate and adaptive immune response. The cells belonging to the innate immunity are neutrophils, which constitute the main component of the inflammatory infiltrate in an intestinal tissue in UC and are one of the first cells participating in the active phase of the disease. Neutrophils recognize phagocytes and take part in incapacitation of the microorganisms through releasing the neutrophil extracellular traps (NETs) or degranulation of its own grains. NETs are cross-linked structures protruding from the membrane of activated neutrophils, composed of condensed chromatin and DNA. Moreover, they contain some components of the neutrophils’ grains, such as neutrophil elastase, myeloperoxidase (MPO), and cathepsin G. NETs generation is a neutrophils’ response to the presence of pathogens since the biochemical composition of NETs determines the trapping and upcoming elimination of pathogens. MPO released from the cell catalyzes the HClO synthesis reaction. Further reactions with this acid affect the formation of reactive oxygen species involved in the inactivation of microorganisms, and thus contribute to tissue damage and ulcer formation [7,8,17,18]. The studies conducted so far show that, in patients with UC, the concentration of MPO was several times higher than the expression of the enzyme in the stool of healthy subjects, which suggests an increased activity of neutrophils in these patients. Factors that increase NET release include tumor necrosis factor-α (TNF-α) and bacterial lipopolysaccharides, although this phenomenon is characteristic for neutrophils’ activation, not only limited to UC. NETs may also arise when stimulated by other pro-inflammatory cytokines, including IL-8 released by endothelial cells, and by NO or neutrophil autoantibodies characteristic to small blood vessel inflammation and also present in UC. Triggering properties towards releasing of NETs also fulfill protein arginine deiminase 4 (PAD4), which is responsible for histone citrullination—a key process taking place during NETosis. Dinallo et al. [17] proved that epithelial cells and cells of intestinal mucosa exhibited higher PAD4 concentrations in intestinal tissue specimens collected from UC patients, compared to healthy individuals and patients with Crohn’s disease. Moreover, comparing the expression of PAD4 in a tissue lesion and in healthy tissue collected from the same patients with UC, it has been noted that in the first case, an expression of PAD4 was significantly higher [7,17,18].

Recently, in pathogenesis of UC, the role of innate lymphoid cells (ILC), which belongs to the family of mononuclear effective cells with common lymphoid progenitor, has been highlighted. The cells take part in the immune response directed towards extra- and intracellular microorganisms, in protection of an intestinal barrier, as well as in tissue repair and remodeling. Taking into account the expression of transcriptional factors and the types of cytokines secreted by the ILC, three types of these cells were distinguished: ILC1, ILC2, ILC3 [5,19,20].

In the Forkel’s et al. [19] analysis, an increased number of ILC1 cells correlated with an early stage of Crohn’s disease. Additionally, Forkel et al. noted a significant increase in the number of ILC1 cells in the inflamed intestinal mucosa. ILC1 appears as a result of enhanced expression of T-cell-specific T-box transcription factor (T-bet), the expression of which is induced by IL-12. ILC1 is mainly responsible for eradication of the viruses and bacteria. In addition, after pathogens are immobilized by DCs that release IL-12 and IL-18, ILC1 is stimulated to synthesize interferon-γ (IFN-γ). Increased secretion of IFN-γ may contribute to the pathological processes seen in UC; however, the role of ILC1 is more emphasized in the pathogenesis of other type of IBD, such as Crohn’s disease, than in UC [5,19,20].

In the chronic stage of UC, the number of ILC1, as well as ILC2 cells, was increased in the intestinal mucosa. Moreover, in biopsies of the inflamed intestinal mucosa collected from the patients with early UC, an increased number of ILC2 cells has also been reported. ILC2 cells appear as a result of enhanced expression of transcription factors such as GATA binding protein 3 (GATA3) and retinoic-related orphan receptor α (RORα). In response to IL-33 (secreted especially during parasite infection, epithelial cells damage or exposition to allergens), ILC2 releases IL-5, responsible for neutrophil recruitment to inflamed areas, and IL-13 that disrupts the intestinal epithelial barrier function. However, ILC2 is also able to secrete IL-4, -6, -8, -9, granulocyte-macrophage colony-stimulating factor (GM-CSF) and amphiregulin, involved in epithelial repair [19,21].

The third, and at the same time the most diversified, group of innate lymphoid cells are the ILC3 cells, which appears as a result of the action of retinoic-related orphan receptor γt (RORγt). Due to the presence of a natural receptor of cytotoxicity, ILC3 cells can be divided into NKp44+ and NKp44− cells. ILC3 cells are well known for secreting IL-22 and/or IL-17 in response to IL-23 and IL-1β [5,19,21]. Patients with IBD present a reduced number of NKp44+ cells, which negatively correlates with disease activity assessed in endoscopic examination. NKp44+ cells are dominant ILC cells in the healthy mucosa of the ileum, caecum and colon. They are characterized by high expression of IL-22 with a protective role towards intestinal epithelial cells, and low expression of IL-17, which may fulfill both protective and pro-inflammatory functions against the intestinal barrier, depending on the cytokine environment. The reduced number of NKp44+ cells may contribute to a reduction in the expression of the protective IL-22 and thus to the dysfunction of the intestinal barrier. Interestingly, no significant differences were found in the ILC population in the peripheral blood and healthy intestinal mucosa from the IBD patients compared to the ILC population in the peripheral blood and intestinal mucosa from healthy people, which indicates the native nature of these changes [5,19,21]. 

### 2.2. Antigen-Presenting Cells (APC)

Antigen-presenting cells, including dendritic cells and macrophages, are cells connecting two types of the immune response. In spite of a different origin, these two types of cells express receptors recognizing molecular patterns such as toll-like receptors (TLR) and nucleotide-binding oligomerization domain-coding protein (NOD). Unlike macrophages, DCs migrate to peripheral lymph nodes when activated, while macrophages locally activate an adaptive immune response. In a healthy organism, intestinal DCs remain immune tolerant because they secrete protective IL-10, while in IBD, DCs shift their activity and the number of pro-inflammatory DCs increases. Hart et al. [22] showed an increased expression of TLR-2 and TLR-4 on the surface of dendritic cells in the biopsy material from patients with UC, while in patients without changes in endoscopic examination, elevated TLR-2 or TLR-4 was found in only a few cases. Activation of these receptors leads to the activation of the nuclear factor kappa-B (NF-κB) and other transcription factors directly influencing the processes related to the development of inflammation [5,20,22].

Macrophages also show significant functional differences depending on the tissue environment. In the absence of inflammatory processes, macrophages perform phagocytic functions, secreting pro-inflammatory cytokines only to a limited extent, and DCs are mainly involved in antigen presentation. During inflammation, the cytokines responsible for macrophages’ activation are secreted and, depending on the method of activation, macrophages can be divided into classically activated (M1) or alternatively activated (M2). M1 present pro-inflammatory functions and significant antibacterial activity. They are activated by exposure to interferon-γ (IFN-γ), GM-CSF or LPS and, when stimulated, secrete significant amounts of cytokines (TNF-α, IL-1β, IL-12, IL-18, IL-23), chemokines (CXCl9, CXCL10), reactive oxygen and nitrogen species. M1 take part in the immune response via Th1 and Th17 cells. In contrast, M2 macrophages induced by IL-4, IL-10, and IL-13 exhibit anti-inflammatory functions and take part in tissue healing and fibrosis. M2 are characterized by the significant expression of mannose receptor (CD206) and scavenger-type receptors (CD163 and CD204), and regulate the activation of Th2 cells through the secretion of IL-10 and TGF-β. In addition, via releasing the anti-inflammatory chemokines (CCL-17, CCL-22, CCL-24), M2 promote basophils and eosinophils recruitment. Intestinal macrophages present features characteristic for M1, as well as M2 cells. On the one hand, similar to M1 cells, they present high expression of antigens belonging to the II class of the major histocompability complex (MHC), along with the expression of TNF-α. On the other hand, like M2 cells, they represent significant phagocytic activity and constitutive expression of IL-10. During IBD, the balance between M1 and M2 is shifted towards the pro-inflammatory type. Intestinal macrophages then secrete pro-inflammatory cytokines, such as IL-6, IL-23 and TNF-α, presenting at the same time increased cytotoxicity and phagocytic activity [1,5]. 

### 2.3. Lymphocytes, as an Element of the Adaptive Immune Response in the UC’s Pathogenesis

**Figure 2:**
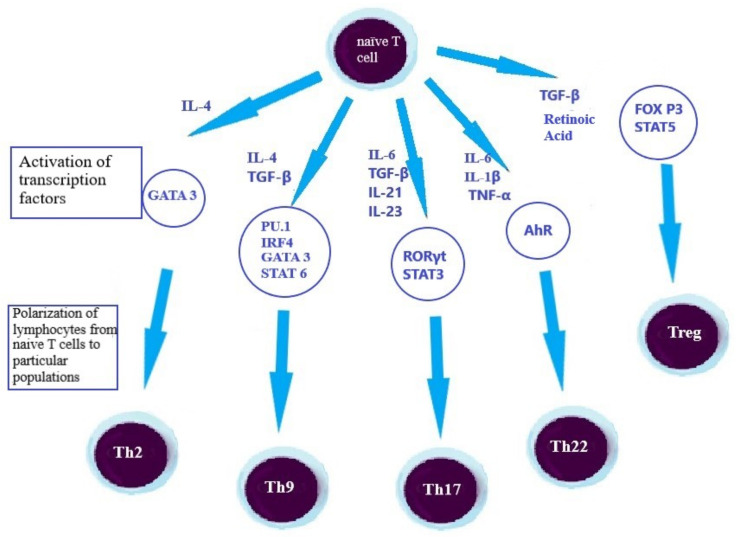
Differentiation of T cells’ main population from the naïve T cells [5,9,25]. AhR—aryl hydrocarbon receptor, FOX P3—forkhead box P3, GATA 3—GATA binding protein 3, IRF4—interferon regulatory factor 4, PU.1—Purine-rich Box-1, RORγt—retinoic acid receptor-related orphan receptor γt, STAT3—signal transducer and activator of transcription 3, STAT5—signal transducer and activator of transcription 5, TGF-β—transforming growth factorβ, TNF-α—tumor necrosis factor α.

Antigen-presenting cells activate the mechanisms of immune response by antigen presentation to T and B cells. This type of immune response is more time-consuming, although it is more precise than the innate immune response. Depending on the expression of the CD4 and CD8 cell surface molecules, lymphocytes can be divided into T CD8+, mainly cytotoxic cells, and T CD4+ T cells. The first type of cells contributes to the pathological changes observed in UC in the intestine through the production of pro-inflammatory cytokines (i.e., IFN-γ, TNF-α) that increase the inflammation and the secretion of pro-inflammatory cytokines and chemokines that damage epithelial cells. These actions of T CD8+ cells cause the formation of ulcers in the intestine that occurs in ulcerative colitis. The latter type, T CD4+ cells, can be subdivided into helper T cells (Th) and regulatory T cells (Treg). Moreover, Th1, Th2, Th9, Th17 and Th22 were distinguished among Th lymphocytes, based on the cytokine profile. Differentiation from naive cells to the aforementioned helper T cell types depends on the cytokine environment and the activation of individual transcription factors, as shown in Figure 2 [5,23,24]. Inflammation within the intestine tissue is associated with an enhanced activation and maturation of T cells. Lymphocytes isolated from the peripheral blood of UC patients showed increased expression of markers of activation—HLA-DR, β1-integrin and decreased expression of CD62L, characteristic for naïve T cells, compared to lymphocytes isolated from healthy individuals. These results indicate an increased activation of CD4+ and CD8+ cells in patients with UC. Moreover, the phenotype of active cells was correlated with an increased level of systemic and intestinal inflammatory markers in newly diagnosed patients. In the same study group, the percentage of inactive T CD4+ cells expressing CD62L negatively correlated with eosinophil cationic protein and calprotectin in feces considered to be intestinal inflammatory markers. Inflammation promotes lymphocyte activation due to increased APC activity in IBD. Thus, the cytotoxicity of T CD8+ cells can lead to tissue damage, which exacerbates the inflammatory processes [24].

### 2.4. Th2 Lymphocytes

Previous research projects have shown that during UC, the expression of Th2 subpopulation of lymphocytes is increased in the inflammatory infiltration of the intestine tissue. Th2 cells are responsible for maintaining the homeostasis of the intestinal mucosa and are also involved in immune response against parasites, pro-inflammatory pathways and tissue repair. Excessive activation of Th2 cells may lead to the development of chronic inflammatory state and progressive tissue fibrosis. Th2 cells mediate the humoral type of immune response, secreting, i.e., IL-4 and IL-10, while by increasing the secretion of IL-5, IL-13, IL-21, and IL-25, they inhibit the activity of Th1 cells involved in cellular immune response [5,26]. Secreted by Th2 cells, IL-13 fulfill a crucial role in the pathogenesis of UC. Through the signal transducer and activator of transcription 6 (STAT-6) and signaling pathways of PI3K kinase, IL-13 influences the integrity of the intestinal barrier, since it increases the expression of claudin-2, creating pores selective for water and small cations. The expression of claudin-2 in the intestinal epithelium is high upon birth, then drops, and the next rise of claudin-2 expression in the intestine epithelium can be observed in inflammatory states including IBD. In addition, IL-13 negatively affects the integrity of the intestinal barrier by inducing apoptosis of intestinal epithelial cells and inhibiting epithelial regeneration. Moreover, TNF-α enhances these effects [24,27,28]. 

Th2 lymphocytes arise from the activation of the GATA-3 transcription factor upon stimulation by IL-4 secreted by dendritic cells. Interestingly, Seidelin et al. [29] presented that intestinal IL-33 is able to increase the expression of GATA-3, which indicates that the increased number of Th2 cells might be caused by an increased level of IL-33. Seidelin et al. noted that the expression of IL-33 was higher in intestinal biopsies collected from patients with active UC compared to inactive UC and healthy subjects. IL-33 plays a dichotomous role in the human body. On the one hand, IL-33 has a protective function as it reduces the expression of genes induced by NF-κB pathway; however, on the other hand, acting as an “alarmin”, IL-33 is constitutively secreted into the extracellular space and impairs immune homeostasis, leading to the development of inflammation. Its role depends, on the degree of damage, the presence of pro-inflammatory cytokines, and the stage and localization of the inflammatory process. IL-33 presents an ability to bind DNA; as a result, it can act as a transcription factor. Additionally, IL-33 is also a conventional cytokine, which binds the ST2 receptor on the cell surface [26,29,30,31,32,33]. Bessa et al. [34] examined the influence of compartmentalization of IL-33 on the inflammation development. The researchers used a mouse model with the IL-33 mutation, which lost nucleus location, and noted that mice with the mutation presented a higher expression of IL-33 and developed a multisystem inflammatory response including, e.g., intestine inflammation. At the same time, no inflammation was noted in the mice without a receptor for IL-33 [34]. Physiologically, IL-33 secretion from the cell occurs as a result of cell damage or apoptosis. IL-33 binds the ST2 receptor present on the surface of Th2, ILC2, Treg, and T CD8+ cells. Activation of IL-33/ST2 axis stimulates the secretion of Th2 cytokines, which enhance the activation of Th2 and ILC2. Via TGF-β, IL-33 stimulates the polarization of naïve lymphocytes into Treg cells and also coordinates the functions of Treg and ILC2 during tissue repair. In UC, the concentration of IL-33 and the soluble ST2 receptor in serum is increased compared to healthy individuals and patients with Crohn’s disease, and additionally correlates with disease activity, which indicates the contribution of the IL-33/ST2 axis in the initiation and/or maintenance of the inflammation [32,33]. 

### 2.5. Th9 Lymphocytes

Other T cells, including Th9, Th17, Th22 and Treg, also play an important role in the pathogenesis of UC. Shohan et al. [10] presented that, in the intestinal biopsies collected from UC patients, a number of Th9 cells was increased compared to the healthy control, which may indicate a crucial role of these cells in UC pathogenesis [10]. Th9 cells appear as a result of polarization of naïve T cells in the presence of IL-4 and TGF-β, thereby acquiring the ability to secrete IL-9. Expression of IL-9 is regulated by various cytokines and transcription factors, such as STAT-6 and GATA-3 (characteristic for Th2 cells), as well as regulatory interferon factor 4 (IRF4) and purine-rich box-1 (PU.1) associated with Th9 cells. In particular, the expression of IL-9 depends on the last of the mentioned factors, since Th9 cells are the main source of IL-9 [25]. IL-9 is involved in the elimination of parasites by activating mast cells and eosinophils. In addition, this interleukin improves mucus synthesis and neutrophil infiltration in an allergic reaction [9]. Moreover, IL-9 also influences the expression of proteins forming the tight junction between the cells, increasing the permeability of the epithelium [10]. 

In the intestinal mucosa of patients with UC, the expression of mRNA encoding IL-9 was increased compared to the intestinal mucosa of healthy individuals; additionally, mRNA expression correlated with disease activity expressed in the Mayo scale. In order to determine the origin of IL-9, the researchers were looking for cells with PU. 1 expression and discovered that, in UC, the quantity of T CD4+ cells with PU. 1 expression was increased compared to the control group. These results may indicate that Th9 cells play a significant role in the UC’s pathogenesis. In the following research, conducted on the mouse model with chronic colitis induced by adoptive T cell transfer, mice with IL-9 deficiency developed less intensified inflammation compared to wild-type mice. Moreover, animals with IL-9 deficiency presented a decreased expression of claudin-2—the protein regulating tight junctions—compared to the wild-type mice, and the rectal administration of recombinant IL-9 increased the expression of claudin-2 in both models of mouse. Thus, IL-9 increases the expression of claudin-2, which, as a selectively permeable pore-forming protein, is one of the factors responsible for the impairment of the intestinal barrier integrity, and promotes the activation of the immune system and increases the progression of colitis. Other factors contributing to intestinal barrier impairment and development of inflammation are the relocation and impaired expression of other proteins, creating tight junctions between epithelial cells (for example, occludin), as well as intensified apoptosis of epithelial cells [25,28,35].

### 2.6. Th17 Lymphocytes

CD4+ T cells in the presence of IL-6, TGF-β, IL-21 and IL-23 evolve into Th17 cells. IL-6 secreted by macrophages and DCs together with TGF-β mediate the activation of RORγt and signal transducer and activators of transcription 3 (STAT3), which are key transcription factors in the polarization of naive T cells to Th17 cells. IL-21 with features similar to IL-6 enhances the expression of the IL-23 receptor, which, by binding to IL-23, induces the polarization of the naive T cells to Th17 cells (by activating the RORγt-dependent pathway), which also affects cells maturation, phenotype and proliferation. Patients with IBD showed increased expression of IL-23 and interleukins belonging to the IL-17 family, which is related to the increased number of Th17 cells [5,20]. Ivanov et al. [36] noted that the induction of Th17 cells may be caused by the adherence of intestinal bacteria (such as segmented filamentous bacterium—SFB) to intestinal epithelial cells. The resulting activation of dendritic cells may lead to secretion of IL-1β and IL-6 in the intestine. The increased level of IL-23, IL-1β and IL-6 causes a polarization of naïve T cells to Th17 cells [36,37]. Th17 cells secrete cytokines such as IL-21, IL-22, IL-12, and TNF-α, as well as IL-17A and IL-17F belonging to the IL-17 family. IL-17A stimulates monocytes, epithelial and endothelial cells to secrete chemokines (CXCL1, CXCL2, CXCL5, CXCL8) that attract lymphocytes and neutrophils to the inflamed tissue. Moreover, IL-17A stimulates the synthesis of pro-inflammatory IL-1β and TNF-α [5,20]. However, Lee et al. [13] noted that the early and IL-23 independent synthesis of IL-17A plays a protective role as it affects the localization of occludin in tight junctions in a mouse model of experimental colitis induced by dextran sodium sulfate. Occludin is a component of a multiprotein complex creating tight junctions between intestinal epithelial cells; therefore, the lack of this protein or its internalization increases the permeability of the intestinal barrier [12,38]. In addition to IL-17, IL-22 also exerts an activity supporting the intestinal barrier function by inducing the synthesis of mucus and antimicrobial peptides [5,21]. Th17 cells play a dual role in human body; on the one hand they secrete pro-inflammatory cytokines (IL-17, TNF-α), and on the other hand, cytokines that protect the intestinal epithelium (IL-10, IL-22). As a result of the excessive activation, Th17 cells may become the pathogenic cells, contributing to chronic inflammation and the development of UC [5,12,20].

### 2.7. Th22 Lymphocytes

Despite the similar functions and phenotype of Th17 cells, Th22 cells show lower expression of RORγt, and polarization from naive T cells to Th22 cells occurs as a result of activation of the aryl hydrocarbon receptor (AhR) by IL-6 and TNF-α [5,39]. Leung et al. [39] noticed that, in UC, the percentage of Th22 cells (CD4+, IL-17−, IL-22+) is reduced, while the percentage of Th17 cells (CD4+, IL-17+, IL-22−) increases in mononuclear cells of the lamina propria of the intestinal mucosa taken during a biopsy. These data indicate a disturbed Th17/Th22 balance in patients with UC. Th22 cells are involved in antiviral, antibacterial and antifungal defense, as well as in the tissue repair within the skin and gastrointestinal tract. These lymphocytes mainly secrete cytokines that protect the intestinal barrier, i.e., IL-10 with immunosuppressive properties, and IL-22, which stimulates epithelial cell proliferation and intestinal mucus synthesis. The analysis [39] performed on the experimental murine models additionally showed that TGF-β is able to inhibit the synthesis of IL-22 via the transcription factor c-Maf. This conclusion was further confirmed in cultures of intestinal lamina propria mononuclear cells taken from healthy individuals, which had been exposed to increasing levels of TGF-β in subsequent cultures. The cultures also contained IL-6 and IL-23, which are necessary for lymphocyte polarization from naive T cells to Th17 and Th22, respectively. High expression of TGF-β suppressed CD4+, IL-17−, and IL-22+ cells, and simultaneously increased the number of CD4+, CD25+, and FoxP3+ Treg cells. These results indicate the suppressive role of TGF-β on Th22 polarization, as well as the pleiotropic impact of this cytokine on lymphocyte polarization in different cytokine environments [5,39,40].

### 2.8. Treg Lymphocytes 

The lymphocytes also expressing CD4+ particles are Treg cells, which provide CD4+—expressed lymphocytes that ensure immune tolerance towards intestine microbiota, as well as preventing the development of autoimmune processes since they secrete anti-inflammatory cytokines such as IL-10, IL-35, and TGF-β. The lack of one of these cytokines, IL-10, secreted by the Treg cells, has been shown to lead to the development of spontaneous colitis in a mouse model. Moreover, in the studies by Woźniak-Stolarska [41], an increased expression of IL-10 was observed in patients with UC. Other researchers have additionally observed that increased expression of IL-10 mRNA correlates with disease activity. The anti-inflammatory effect of IL-10 is associated with inhibition of NF-κB, tissue factor, tissue metalloproteinases, and cyclooxygenase 2 [5,11,42]. TGF-β, similarly to IL-10, fulfills the immunosuppressive role. Research conducted on the IL-6 knock-out mouse model presented an elevated level of Treg cells and decreased level of Th17 cells in these mice. TGF-β in the absence of IL-6 suppresses Th17 polarization from naïve T cells and thus, via RORγt, stimulates Treg polarization. In addition, Treg cells may suppress polarization of Th17 cells through IL-10 and IL-35, as well suppressing secretion of cytokines activating Th17 differentiation (IL-6, IL-23) [12]. Treg cells through TGF-β stimulate tissue repair and relieve inflammation by suppressing the expression of IL-33 receptor [13]. Patients with UC, compared to healthy individuals, present an increased number of Treg cells; however, these cells are more likely to undergo apoptosis in inflamed tissue and show a 60% reduced ability to suppress proliferation of other T cells [5].

### 2.9. B Lymphocytes/Plasmocytes

In UC pathogenesis, B lymphocytes play a crucial role beside T cells. B cells are responsible for the synthesis of antibodies, antigen presentation to T cells and adaptation of the inflammatory response through secretion of IL-2, IL-4, IFN-γ, TGF-β and GM-CSF. Regarding the function of the cell, B cells may be divided into effector cells—secreting antibodies and cytokines—and regulatory cells (Breg), secreting IL-10. Activation of B cells in the intestines takes place in mesenteric lymph nodes and lymph nodules, followed by the migration from nodes and nodules to the intestinal lamina propria and differentiation into plasmocytes, which is associated with the disappearance of the CD19 antigen [43,44]. 

B cell surface markers have not been clearly identified; however, it is known that CD5 antigen is located on the surface of regulatory lymphocytes. The studies performed showed that Breg cells transformed to plasmocytes can highly express CD24 and CD38 (CD24^high^ CD38^high^). Moreover, the expression of CD95 antigen on the surface of Breg indicates functional exhaustion of B cells, which can be followed by a loss of its functions. Wang et al. [45] used flow cytometry to distinguish the expression of various B-cell surface markers in the peripheral blood of IBD patients. Patients with UC presented a decreased expression of cells with CD24^high^ CD38^high^ and CD5+ phenotypes, compared to healthy individuals. In the course of UC, a reduction of Breg cell population is observed, which leads to a decrease in the concentration of IL-10 in the peripheral blood. Furthermore, the percentage of CD24^high^ CD38^high^ cells negatively correlates with disease activity based on the Mayo scale. The highest percentage of CD95+ cells was found among CD5+ Breg cells in UC patients, compared to healthy subjects, and this percentage also positively correlated with the Mayo scale. The ability of B cells isolated from UC patients to secrete IL-10 as a result of LPS and CD257 stimulation was evaluated, and it was found that the majority of IL-10 secreting cells were those with CD24^high^ CD38^high^ CD5+. However, the reduction in the population of these cells resulted in a decrease in IL-10 levels in UC patients compared with healthy individuals. Thus, patients with UC may present a decreased quantity of B cells, which perform regulatory functions by secreting IL-10 with immunosuppressive properties. At the same time, Breg cells show a reduced functional activity, as evidenced by high expression of the CD95+ antigen. These findings might suggest that the pathogenesis of UC is connected with paucity of regulatory B cells and their decreased immunoregulatory properties. At the same time, these results may provide a new potential approach to UC treatment [45]. 

Plasmocytes are the terminal stage of B cell differentiation and are localized mainly in bone marrow and lamina propria. IgA-secreting cells represent the majority of the intestinal plasmocytes, while IgG-secreting cells are related to inflammation. In the UC, the number of plasmocytes is elevated in the intestinal tissue and the balance between these two types of cells is changed, which indicates a significant role of these cells in the pathogenesis of UC. Research conducted by Uo et al. [46] confirms these reports, as the immunohistochemistry examination of UC’s mucosal presented a significant influx of plasmocytes to the intestinal mucosa. Moreover, the majority of plasmocytes isolated from the non-IBD controls secrete IgA, while in the UC, plasmocytes secrete mostly IgG [41]. Under physiological conditions, locally secreted antibodies regulate the composition of the microbiota composition and maintain the intestinal barrier homeostasis. In UC, the synthesis of antibodies against one’s own microbiota or tissues such as antibodies to flagella or perinuclear antibodies to neutrophils, is frequently observed, and is detected in 2/3 of UC patients. Castro-Dopico et al. [47] assessed the degree of binding of intestinal microbiota by IgG antibodies in the feces. It has been shown that, in UC, the opsonization of gut bacteria by antibodies is increased compared to healthy individuals. Eighty percent of the bacteria isolated from the feces of UC patients were opsonized by antibodies, while in healthy controls less than 10% of the bacteria were found to be opsonized [44,47,48]. 

As mentioned above, the IgG is mainly secreted in the inflammation and is the main immunoglobulin present in the intestinal tissue in UC. Class G immunoglobulins show high affinity to antigens, the ability to activate the complement system by binding C1q, and also are responsible for cellular memory. By binding to FcγR, IgG eliminates microorganisms, influences migration and maturation of immune cells, and affects the synthesis of inflammatory mediators. FcγR are glycoproteins expressed on the surface of immune cells and can act as activators (FcγRI, FcγRIIA, FcγRIIIA, FcγRIIIB) or inhibitors (FcγRIIB) of the immune response. FcγRs bind mainly to IgG, but can also bind serum amyloid P or C-reactive protein (CRP). In particular, the relationship between CRP and FcγRIIA causes the internalization of autoantigens, microorganisms and damaged cells by neutrophils and monocytes. In UC, the neutrophils state the main component of the intestinal infiltration, which can be associated with the increased expression of genes coding IL-1β, CXCL1 and CXCL8, responsible for recruitment of neutrophils. Expression of IL-1β and CXCL8 correlates most with FcγRIIA expression, which may indicate that IgG antibodies against gut microbiota contribute to the development of intestinal inflammation through FcγRIIA-dependent induction of IL-1β and CXCL8. Among patients with IBD, the cases of incorrect post-translational modification were noted, including defucosylation, enhanced affinity of IgG to FcγRIIA or agalactosylation, and decreased affinity of IgG to FCγRIIB [47,48].

**Figure 3:**
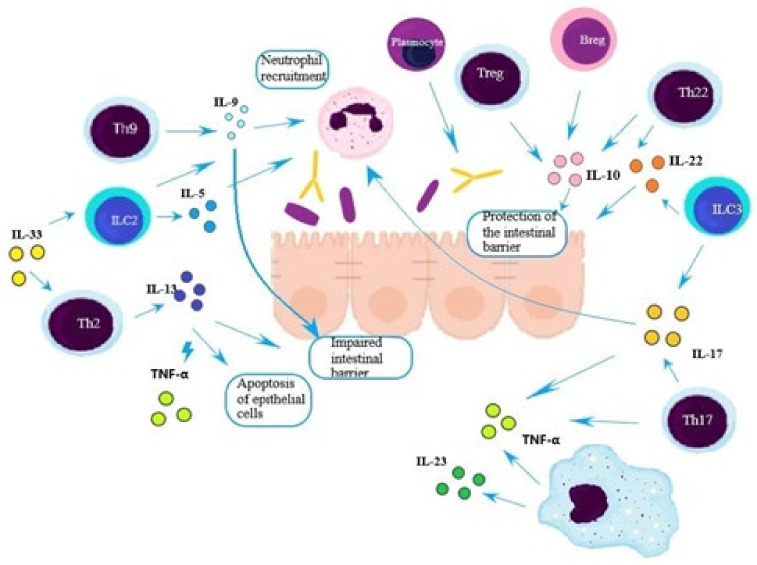
The role of immune cells in the development of ulcerative colitis (UC). Among patients with UC, an increased number of Th2, Th9, and Th17 lymphocytes, as well as ILC2 and ILC3, is observed. The cytokines secreted by these cells contribute to the impairment of the intestinal barrier function through increased expression of claudin-2 and increased apoptosis of epithelial cells. Additionally, cytokines stimulate the migration and degranulation of neutrophils and further activation of immune cells. Activated plasma cells secrete antibodies, including those against gut microbiota inducing chemokines and IL-1β [5,27,33,45]. At the same time, the number and/or activity of Breg, Treg, and Th22 cells is decreased, which leads to reduced protection of the intestinal barrier [5,11,42,45].

The role of innate cells in the development of ulcerative colitis has been summarized in Figure 3.

## 3. Conclusions

Ulcerative colitis is associated with chronic inflammation, which possibly results from the abnormal immune response towards intestine microbiota and/or food antigens. The exact mechanisms contributing to the development of UC remain unclear, but significant progress has been made recently, including a better understanding of the innate functions of lymphoid cells and their relationship to changes in disease development. Moreover, Th9 cells were identified among lymphocytes, which by secreting IL-9 influence the integrity of the intestinal barrier and contribute to the degranulation of neutrophils. Among patients with UC, increased activity was noted, as well as the number of pro-inflammatory Th2, Th17, and Th9, with simultaneous inhibition of the activity and number of anti-inflammatory cells—Th22, Treg, and Breg. The activity of immune cells is affected by the cytokines. Cytokines not only contribute to the development and maintenance of inflammation, but also influence the integrity of the intestinal barrier. IL-10 and IL-22 protect the intestinal barrier, while IL-13 and IL-9 disrupt the intestinal barrier by increasing the expression of claudin-2 in tight junctions between epithelial cells and enhancing the apoptosis of the intestinal barrier’s epithelial cells. Interestingly, the functions of the cytokines are depended on the cytokine environment; for example, TNF-α enhances the effect of IL-13. The above relationship may be useful in the treatment of UC, because the therapeutic effect of eliminating the influence of one cytokine on the disease process may be canceled out by another type of cytokine.

## Figures and Tables

**Table 1:** The influence of cytokines taking part in the UC pathogenesis on the development of the immune response [5,7,8,9,10,11,12,13,14,15,16].

Cytokine	Expression of Cytokine in UC	Cells Secreting Cytokine	Influence on the Inflammation	Function
IL-1β	Increased	Macrophages	pro-inflammatory	Stimulates T cells to secrete pro-inflammatory cytokines, induces chemotactic factors, influences the proliferation of B cells, systemically causes an increase of temperature and acute phase proteins
IL-4		Th2	anti-inflammatory	Stimulates humoral immune response, inhibits the secretion of pro-inflammatory cytokines (TNF-α, IL-6, IL-1β), activates M2 macrophages, activates transcription factors responsible for differentiation of T CD4+ cells to Th9 cells
IL-5	Increased	Th2		Decreases the activity of Th1 cells and cellular immune response, stimulates the maturation of eosinophils and basophils
IL-6	Increased	Macrophages, dendritic cells	pro-inflammatory	Activates transcription factors responsible for differentiation of T CD4+ cells to Th17 cells, inhibits the secretion of TNF-α
IL-9	Increased	Th9, Th2	pro-inflammatory	Activates mast cells, neutrophils and eosinophils, influences the expression of proteins creating tight junctions in the intestinal barrier
IL-10	Increased	Treg, Th2, Th17, Breg	anti-inflammatory	Inhibits the secretion of tissue metalloproteinases, tissue factor and cyclooxygenase 2, suppresses the expression of transcription factor NF-κB, activates macrophages M2
IL-13	Increased	Th2	anti-inflammatory	Decreases the activity of Th1 cells and cellular immune response, inhibits the secretion of pro-inflammatory cytokines (TNF-α, IL-6, IL-1β), influences the expression of proteins creating tight junctions in the intestinal barrier and the epithelial cells apoptosis, activates macrophages M2
IL-17	Increased	Th17, monocytes, neutrophils, T CD8+, NK cells	pro-inflammatory	Stimulates monocytes, epithelial and endothelial cells to secrete pro-inflammatory cytokines (IL-1β, TNF-α) and chemokines responsible for leukocytes and neutrophils migration to inflamed tissues, in the absence of IL-23 supports the intestinal barrier through occludin regulation in tight junctions
IL-21		Th17, Th2		Decreases the activity of Th1 cells and thus the cellular immune response, increases the expression of IL-23 receptor, stimulates proliferation and maturation of B, T CD8+ and NK cells
IL-22		Th17, Th22, Th1		Induces the secretion of antimicrobial peptides, IL-10 and mucus, mediates in the tissue repair processes
IL-23	Increased	Macrophages, dendritic cells	pro-inflammatory	Takes part in the differentiation of T CD4+ cells to Th17 cells
IL-25		Th2		Decreases the activity of Th1 cells and cellular immune response
IL-33		Treg, macrophages, dendritic cells, mast cells		Enhances the secretion of IL-4, IL-5, IL-13, increases the accumulation of Th2, stimulates pathogenic Th2 and Th17 response, induces tissue repair through coordination of Treg
IL-35		Treg	anti-inflammatory	Suppress the differentiation of Th17
TGF-β		Treg	anti-inflammatory	Stimulates epithelial repair, decreases expression of IL-33 and Th22, stimulates differentiation of Th17 in the presence of IL-6 and Treg in the absence of IL-6, activates the transcription factors responsible for differentiation of T CD4+ cells to Th9 cells
IFN-γ		Th1, dendritic cells, macrophages		Enhances transcytosis and paracellular transport, activates macrophages
TNF-α	Increased	Th17, macrophages, dendritic cells	pro-inflammatory	Takes part in cell apoptosis, stimulates lymphocytes and activates other immune cells
